# Weight change following diagnosis with psychosis: a retrospective cohort study in Greater Manchester, UK

**DOI:** 10.1186/s12991-023-00485-8

**Published:** 2024-01-03

**Authors:** Adrian Heald, Chris Daly, John Julian Warner-Levy, Richard Williams, Cheyenne Meehan, Mark Livingston, Toby Pillinger, Lamiece Hussain, Joseph Firth

**Affiliations:** 1 The School of Medicine and Manchester Academic Health Sciences Centre, Manchester University, Manchester, UK; 2 Department of Endocrinology and Diabetes, Salford Royal Hospital, Salford, M6 8HD UK; 3 Greater Manchester Mental Health, Prestwich Hospital, Greater Manchester, UK; 4 Division of Informatics, Imaging and Data Science, Faculty of Biology, Medicine and Health, University of Manchester, Manchester Academic Health Science Centre, Manchester, UK; 5 Black CountryPathology Services, West Midlands, UK; 6 Institute of Psychiatry, De Crespigny Park, London, UK; 7 Division of Psychology and Mental Health, University of Manchester, Manchester Academic Health Science Centre, Manchester, UK

**Keywords:** Non-affective psychosis, Affective psychosis, Longitudinal, Weight gain, BMI

## Abstract

**Introduction:**

Weight gain in the months/years after diagnosis/treatment of severe enduring mental illness (SMI) is a major predictor of future diabetes, dysmetabolic profile and increased risk of cardiometabolic diseases. There is limited data on the longer-term profile of weight change in people with a history of SMI and how this may differ between individuals. We here report a retrospective study on weight change over the 5 years following an SMI diagnosis in Greater Manchester UK, an ethnically and culturally diverse community, with particular focus on comparing non-affective psychosis (NAP) vs affective psychosis (AP) diagnoses.

**Methods:**

We undertook an anonymised search in the Greater Manchester Care Record (GMCR). We reviewed the health records of anyone who had been diagnosed for the first time with first episode psychosis, schizophrenia, schizoaffective disorder, delusional disorder (non-affective psychosis = NAP) or affective psychosis (AP). We analysed body mass index (BMI) change in the 5-year period following the first prescription of antipsychotic medication. All individuals had taken an antipsychotic agent for at least 3 months. The 5-year follow-up point was anywhere between 2003 and 2023.

**Results:**

We identified 9125 people with the diagnoses above. NAP (*n =* 5618; 37.3% female) mean age 49.9 years; AP (*n =* 4131; 60.5% female) mean age 48.7 years. 27.0% of NAP were of non-White ethnicity vs 17.8% of AP individuals. A higher proportion of people diagnosed with NAP were in the highest quintile of social disadvantage 52.4% vs 39.5% for AP. There were no significant differences in baseline BMI profile. In a subsample with HbA1c data (*n =* 2103), mean HbA1c was higher in NAP at baseline (40.4 mmol/mol in NAP vs 36.7 mmol/mol for AP). At 5-year follow-up, there was similarity in both the overall % of individuals in the obese ≥ 30 kg/m^2^ category (39.8% NAP vs 39.7% AP), and % progressing from a normal healthy BMI transitioned to obese/overweight BMI (53.6% of NAP vs 55.6% with AP). 43.7% of those NAP with normal BMI remained at a healthy BMI vs 42.7% with AP. At 5-year follow-up for NAP, 83.1% of those with BMI ≥ 30 kg/m^2^ stayed in this category vs 81.5% of AP.

**Conclusion:**

The results of this real-world longitudinal cohort study suggest that the changes in BMI with treatment of non-affective psychosis vs bipolar disorder are not significantly different, while 43% maintain a healthy weight in the first 5 years following antipsychotic prescription.

## Key summary

We here report a longitudinal retrospective cohort study on weight change post-SMI diagnosis in Greater Manchester UK, an ethnically and culturally diverse community with particular focus on a history of non-affective psychosis vs affective psychosis disorder.

## Introduction

Weight gain in relation to treatment of major mental illness is a determinant of future diabetes, dysmetabolic profile and increased cardiometabolic risk in people treated with antipsychotic agents [1, 2]. Early weight gain is a predictor of longer-term weight gain, with the attendant long-term consequences including premature cardiovascular events and death [3]. Genetic factors likely play a significant part in the degree to which weight gain occurs [4].

Considerable variability in weight gain and metabolic effects exists between individuals in both the intermediate and longer term [5]. Young and antipsychotic-naïve patients are at particularly high risk of weight gain in the short to intermediate term [6]. Many factors contribute to weight gain in patients with schizophrenia or psychosis. Of these, sedentary lifestyle, unhealthy food habits, genetic susceptibility and antipsychotic treatment are considered the main contributors [7].

The matter of predicting who is going to put on weight/increase body mass index (BMI) following initiation of antipsychotic treatment remains a major issue for health care professionals working in psychiatry, and also for service users. At present, risk prediction models are imperfect [8]. There is limited data on the longer-term profile of weight change in people with a history of SMI and how this may differ between individuals. A question related to this is whether there is a difference in weight change over time between people with affective vs non-affective psychosis, given the likely different underlying pathophysiology.

**Fig. 1:**
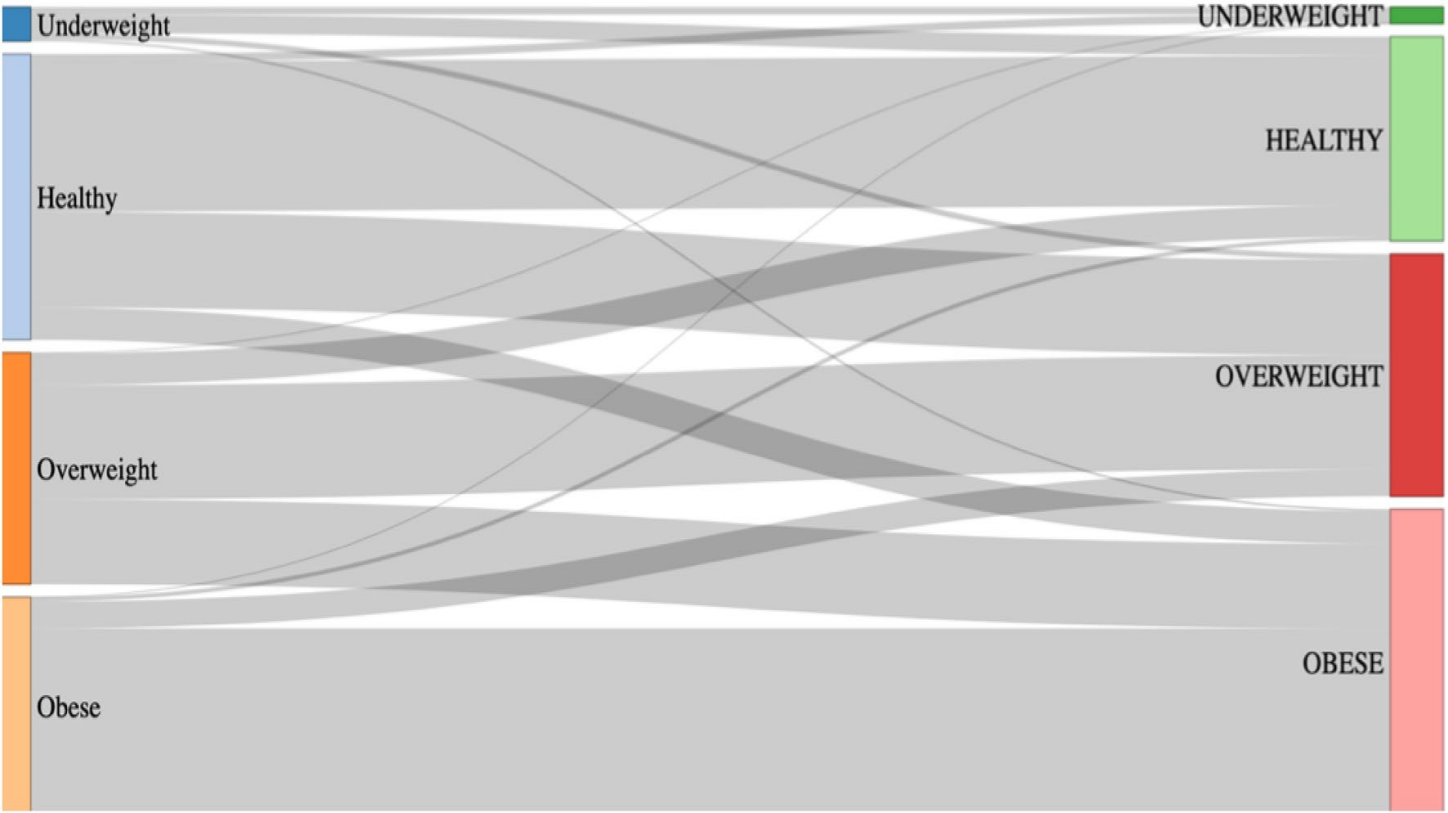
Sankey plot showing the proportion of people transitioning between BMI categories between baseline and 5-year follow-up

We here report a 25-year perspective on weight change post-SMI diagnosis in Greater Manchester, UK, an ethnically and demographically diverse community, with particular focus on a history of psychosis vs bipolar affective disorder (see Fig. 1).

## Materials and methods

We undertook an anonymised search using the Greater Manchester Care Record (GMCR) database. The GMCR is an integrated database of primary care, secondary care and mental health trusts from across Greater Manchester (https://gmwearebettertogether.com/research-and-planning/) [9] for retrospective analyses covering a population of approximately 3 million people. Health and care data were collected from 433 of 435 (99.5%) general practices in GM. Data were de-identified at source and were extracted from the GMCR database.

This project was reviewed, and ethical approval was overseen by Health Innovation Manchester and granted by the Greater Manchester Care Record (GMCR) [9] board (ref: IDCR-RQ-036). This research was performed with anonymised data, in line with the Health Research Authority’s Governance arrangements for research ethics committees.

We reviewed the health records of anyone aged 18 years or over who had been diagnosed for the first time over a 25-year period between 1st January 1997 and 1st January 2018 with first episode psychosis, schizophrenia, schizoaffective disorder, delusional disorder (non-affective psychosis, NAP), psychosis associated with depression or bipolar affective disorder (affective psychosis, AP). We examined the body mass index (BMI) in the period before and after first prescription of antipsychotic medication. The 5-year follow-up period was anywhere between 1998 and 2023.

The sample was restricted to people who were prescribed an antipsychotic for a continuous period (at any time in the five years after diagnosis) for 3 months or more and had at least one BMI measurement prior to the first antipsychotic prescribed date (closest measure) and had another BMI measurement after the first antipsychotic prescription date. We also excluded BMI and antipsychotic observations that had an implausible BMI measurement (< 12 or > 70). People with dementia as a diagnosis were excluded. This left 9,125 people and 92,895 measurements of BMI out of a total of 11,300 with NAP and 6667 people with AP (total 17,967 people).

We conducted a multiple linear regression analysis to investigate the relation between BMI change over time and potentially related factors. The exact numbers in each analysis differed slightly in relation to the specific analysis conducted.

## Results

We identified 9125 people with the diagnoses above. NAP (*n =* 5618; 37.3% female) mean age 49.9 years; AP (*n =* 4131; 60.5% female) mean age 48.7 years. We excluded those people with no follow-up BMI data within the first 6 weeks of initial diagnosis.

Baseline characteristics (relating to the closest point to the first diagnosis of psychosis) are given in Table 1. Diagnosed hypertension was similar in the two groups, although with a higher proportion of people reported as current or ex-smokers at 66.2% in the NAP group vs 62.6% for the AP group. 27.0% of NAP were of non-white ethnicity vs 17.8% of AP individuals.

**Table 1:** Baseline characteristics

Diagnosis	*N*	Female (%)	Mean Age (years)	COPD (%)	Asthma (%)	Hyper-tension (%)	Current/ex-smoker (%)
Psychosis/schizophrenia	5618	37.3	49.9	6.9	17.7	20.3	64.2
Affective psychosis	4131	60.5	48.7	6.0	22.1	19.3	62.6

A higher proportion of people diagnosed with NAP were in the highest quintile of social disadvantage (52.4% NAP vs 39.5% for AP). There were no significant differences in baseline BMI profile between NAP and AP individuals (Table 2) but for those 2103 people where HbA1c was available at baseline, mean HbA1c was higher in NAP at 40.4 mmol/mol vs 36.7 mmol/mol for AP. At 5-year follow-up, the proportion of overweight individuals (BMI 25.0–29.9 kg/m^2^) was slightly lower in NAP at 30.9% (vs 32.3% on AP), while the proportion of obese individuals (BMI 30 kg/m^2^ or more) was similar (39.8% in NAP vs 39.7% in AP) (Table 2).

**Table 2:** BMI at baseline by category

BMI category (kg/m^2^)	Non-affective psychosis		Affective psychosis	
	*N (people)*	*%*	*N (people)*	*%*
Underweight (< 18.5)	299	5.3	148	3.6
Healthy weight (18.5–25)	2267	40.4	1641	39.7
Overweight (25–29.9)	1659	29.5	1265	30.6
Obese (30 or more)	1393	24.8	1077	26.1
BMI at follow-up (5 years) by category				
Underweight (< 18.5)	90	2.2	59	1.9
Healthy weight (18.5–25)	1102	27.1	798	26.1
Overweight (25–29.9)	1256	30.9	986	32.3
Obese (30 or more)	1621	39.8	1212	39.7

The change in BMI category between baseline and 5-year follow-up is shown in Fig. 1. At 5-year follow-up, 53.6% of those NAP with a healthy BMI (18.5–25.0 kg/m^2^) transitioned to obese/overweight BMI, vs 55.6% with AP. 43.7% of those NAP with healthy BMI at baseline remained at a healthy BMI, vs 42.7% with AP. At 5-year follow-up for NAP, 83.1% of those with BMI ≥ 30 kg/m^2^ stayed in this category, vs 81.5% of AP (Table 3).

**Table 3:** BMI category change from baseline to 5-year follow-up

Pre	Post	*n*	Percent
Schizophrenia/psychosis (*N* = 3189)			
Underweight	Healthy	90	2.82
Underweight	Morbidly obese	5	0.16
Underweight	Obese	10	0.31
Underweight	Overweight	32	1.00
Underweight	Underweight	35	1.10
Healthy	Healthy	514	16.12
Healthy	Morbidly obese	57	1.79
Healthy	Obese	152	4.77
Healthy	Overweight	421	13.20
Healthy	Underweight	32	1.00
Overweight	Healthy	133	4.17
Overweight	Morbidly obese	97	3.04
Overweight	Obese	305	9.56
Overweight	Overweight	431	13.52
Overweight	Underweight	7	0.22
Obese	Healthy	15	0.47
Obese	Morbidly obese	154	4.83
Obese	Obese	223	6.99
Obese	Overweight	104	3.26
Morbidly obese	Healthy	5	0.16
Morbidly obese	Morbidly obese	280	8.78
Morbidly obese	Obese	68	2.13
Morbidly obese	Overweight	23	0.72
Bipolar disorder (N = 2373)			
Underweight	Healthy	45	1.90
Underweight	Morbidly obese	5	0.21
Underweight	Obese	5	0.21
Underweight	Overweight	9	0.38
Underweight	Underweight	15	0.63
Healthy	Healthy	380	16.01
Healthy	Morbidly obese	35	1.47
Healthy	Obese	107	4.51
Healthy	Overweight	352	14.83
Healthy	Underweight	15	0.63
Overweight	Healthy	88	3.71
Overweight	Morbidly obese	102	4.30
Overweight	Obese	241	10.16
Overweight	Overweight	309	13.02
Overweight	Underweight	5	0.21
Obese	Healthy	14	0.59
Obese	Morbidly obese	149	6.28
Obese	Obese	164	6.91
Obese	Overweight	78	3.29
Obese	Underweight	5	0.21
Morbidly obese	Healthy	5	0.21
Morbidly obese	Morbidly obese	202	8.51
Morbidly obese	Obese	37	1.56
Morbidly obese	Overweight	18	0.76
Morbidly obese	Underweight	5	0.21

18.6% who were underweight with NAP (BMI < 18.5 kg/m^2^) remained underweight vs 19.0% with AP. 74.5% who were morbidly obese (BMI 40 kg/m^2^ or more) with NAP remained morbidly obese, vs 77.1 of AP individuals.

For those people for whom there was BMI data beyond 10 years (total 3045 individuals combined with NAP and AP), there was further transition of 5% of those with obesity to the morbidly obese category and of 10% of those in the overweight to the obese/morbidly obese category with 12% of normal BMI individuals transitioning to overweight or obese by > 10 years after baseline.

### Linear regression analysis results

Increase in weight of 2 kg or more over the 5-year follow-up period was related to lower initial BMI (*r*^2^ = 0.13, *p* = 0.009), female sex (*r*^2^ = 0.16, *p* = 0.008), younger age at diagnosis (*r*^2^ = 0.10, *p* = 0.01), non-White ethnicity (*r*^2^ = 0.11, *p* = 0.015), independent of NAP vs AP category.

## Discussion

The results of this real-world longitudinal cohort study suggest that the changes in BMI with treatment of non-affective psychosis vs bipolar disorder are not significantly different, highlighting the importance of regular physical health monitoring in all people treated with antipsychotics, not just those with a diagnosis of schizophrenia, while also suggesting an avenue for future work in this area in terms of providing insight into the proportions of people who remain in the same BMI category vs those who change BMI category.

The choice of antipsychotic agent has multiple influences, with polypharmacy likely a factor [5, 10–12]. Importantly the period that we were looking at corresponds with the transition from typical to atypical antipsychotic prescribing in relation to routine prescribing practice in the United Kingdom (UK) [13, 14]. We previously reported a tendency to increasing polypharmacy over time in relation to prescribing for people with a diagnosis of psychosis [15]. Although we recently reported a difference in BMI change between NAP and AP individuals, this was in a much smaller cohort in the setting of one primary care network in England [15].

There is evidence that significant weight gain affects concordance with psychotropic medication [16]. A study by Weiden et al. found that patients who are obese are 13 times more likely to discontinue medication because of weight gain than nonobese patients [17]. This was reported in the CATIE study as well, where more individuals discontinued olanzapine due to weight gain compared to other medications, despite olanzapine showing the lowest overall discontinuation rate [18, 19].

On the other hand, Kinon et al. [20] observed that acute weight gain may be an indicator of better response to antipsychotics and concordance can be expected to improve with improvement in mental health. In that study, weight gain during olanzapine treatment trended toward a plateau after the first 39 weeks of treatment, with no further significant gain up to 3 years. A recent study investigating factors associated with less-than-ideal concordance in people with bipolar disorder reported no difference in adherence between weight groups [21]. Of relevance, the expert consensus guideline by Velligan et al. regarding medication adherence of patients with serious psychiatric illness identified weight gain as a likely factor leading to non-concordance [22]. The consensus stated that is important to identify the specific factors that may be contributing to a patient’s concordance challenges, in order to customize interventions to target those problems.

The psychosocial consequences of weight gain include demoralisation, physical discomfort, and social stigma [23]. In the study by Weiden et al. [17], subjective distress over weight gain was found to be the primary mediator of non-concordance. In a related qualitative study, interviews of 63 first episode patients aged 14–35 years showed that a change in self-identity ensued with the change in physical appearance resulting from weight gain [24].

In this study, an increase of 2 kg or more over the 5-year follow-up period was related to lower initial BMI, younger age at diagnosis, non-White ethnicity, finding which are in accordance with previous studies [25–27].

The ability to identify which patients are at risk of initial weight gain would be invaluable knowledge for clinicians, enabling more focus on preventing initial weight gain [28, 29]. While we are not able to look at risk/protective factors here, we feel that the patterns of transition over time seen with affective and non-affective psychosis in relation to propensity to put on weight after initiation of antipsychotic medication may inform further research in this area.

Given the close links between obesity/overweight and the development of type 2 diabetes (T2D) in people treated with antipsychotic agents [30] with the associated increase in cardiovascular disease [31], greater understanding of the factors linked to weight change over time will benefit patients and inform clinicians. Putative mechanisms of antipsychotic-related weight gain include poor diet and reduced physical activity due to negative symptoms [32]. The interaction between specific antipsychotic prescribing and weight change over time will be the subject of further work on this cohort.

Over the years of follow-up of the individuals in this cohort, there has also been a change in the background population BMI profile across the United Kingdom population. We accept that the changes reported here do reflect that—nevertheless the proportions of people at 5-year follow-up in the obese and morbidly obese categories are higher than seen in the general population.

## Strengths/limitations

We have been able to access real-world data on people diagnosed with psychosis. Importantly the data were extracted from a population database covering 2.85 million people living in an ethnically and culturally diverse European conurbation. However, not everyone with a diagnosis of psychosis underwent sufficient BMI measurements to enable any analysis of weight change over time, illustrating the continuing challenge in the United Kingdom (UK) and elsewhere to ensure that people being treated for psychosis undergo regular physical health checks including weight. Another limitation is that we were not privy to whether the individual had any family history of obesity. Importantly, we were unable to examine different lengths and types of prescription at this stage—and these limitations may account for the relatively low frequency of changes in BMI compared to previous meta-analysis on drug-induced weight gain following antipsychotic initiation [30, 33]. Finally, we accept that in such a real-world study as this, primary care follow-up data may be sparse because of the challenges inherent in facilitating regular physical health checks.

## Conclusion

The results of this longitudinal cohort study using data collected over 25 years in real-world settings suggest that the changes in BMI with treatment of non-affective psychosis vs bipolar disorder are not significantly different following the first 5 years of antipsychotic treatment, while around 43% of people maintain a healthy weight over time.

## Data Availability

The data that support the findings of this study are available from the corresponding author upon reasonable request.

## References

[1] Heald AH, Martin JL, Payton T, Khalid L, Anderson SG, Narayanan RP, De Hert M, Yung A, Livingston M (2017). Changes in metabolic parameters in patients with severe mental illness over a 10-year period: a retrospective cohort study. Aust NZ J Psychiatry..

[2] Zhang JP, Gallego JA, Robinson DG, Malhotra AK, Kane JM, Correll CU (2013). Efficacy and safety of individual second-generation vs. first-generation antipsychotics in first-episode psychosis: a systematic review and meta-analysis. Int J Neuropsychopharmacol.

[3] Allison DB, Mentore JL, Heo M, Chandler LP, Cappelleri JC, Infante MC, Weiden PJ (1999). Antipsychotic-induced weight gain: a comprehensive research synthesis. Am J Psychiatry.

[4] Brandl EJ, Kennedy JL, Müller DJ (2014). Pharmacogenetics of antipsychotics. Can J Psychiatry.

[5] Cooper SJ, Reynolds GP, Barnes T, England E, Haddad PM, Heald A, Holt R, Lingford-Hughes A, Osborn D, McGowan O, Patel MX, Paton C, Reid P, Shiers D, Smith J, With expert co-authors (in alphabetical order) (2016). BAP guidelines on the management of weight gain, metabolic disturbances and cardiovascular risk associated with psychosis and antipsychotic drug treatment. J Psychopharmacol.

[6] Correll CU, Robinson DG, Schooler NR, Brunette MF, Mueser KT, Rosenheck RA (2014). Cardiometabolic risk in patients with first-episode schizophrenia spectrum disorders: baseline results from the RAISE-ETP study. JAMA Psychiat.

[7] Shrivastava A, Johnston ME (2010). Weight-gain in psychiatric treatment: risks, implications, and strategies for prevention and management. Mens Sana Monogr.

[8] Harrison RNS, Gaughran F, Murray RM, Lee SH, Cano JP, Dempster D, Curtis CJ, Dima D, Patel H, de Jong S, Breen G (2017). Development of multivariable models to predict change in Body Mass Index within a clinical trial population of psychotic individuals. Sci Rep.

[9] https://gmwearebettertogether.com/research-and-planning/. Accessed 3 July 2023.

[10] Campforts B, Drukker M, Crins J, van Amelsvoort T, Bak M (2023). Association between antipsychotic medication and clinically relevant weight change: meta-analysis. BJPsych Open.

[11] Anderson SG, Livingston M, Couchman L, Smith DJ, Connolly M, Miller J, Flanagan RJ, Heald AH (2015). Sex differences in plasma clozapine and norclozapine concentrations in clinical practice and in relation to body mass index and plasma glucose concentrations: a retrospective survey. Ann Gen Psychiatry.

[12] Heald A, Azadbakht N, Geary B, Conen S, Fachim H, Lee DCH, Geifman N, Farman S, Howes O, Whetton A, Deakin B (2020). Application of SWATH mass spectrometry in the identification of circulating proteins does not predict future weight gain in early psychosis. Clin Proteomics.

[13] Heald AH, Stedman M, Farman S, Khine C, Davies M, De Hert M, Taylor D (2020). Links between the amount of antipsychotic medication prescribed per population at general practice level, local demographic factors and medication selection. BMC Psychiatry.

[14] Heald A, Livingston M, Yung A, De Hert MA (2017). Prescribing in schizophrenia and psychosis: Increasing polypharmacy over time. Hum Psychopharmacol.

[15] Remington G, Hahn MK, Agarwal SM, Chintoh A, Agid O (2021). Schizophrenia: antipsychotics and drug development. Behav Brain Res.

[16] Roerig JL, Steffen KJ, Mitchell JE (2011). Atypical antipsychotic-induced weight gain: insights into mechanisms of action. CNS Drugs.

[17] Heald AH, Stedman M, Daly C, Warner-Levy JJ, Livingston M, Hussain L, Anderson S (2023). First episode psychosis and weight gain a longitudinal perspective in Cheshire UK: a comparison between individuals with nonaffective versus affective psychosis. Cardiovasc Endocrinol Metab.

[18] Dayabandara M, Hanwella R, Ratnatunga S, Seneviratne S, Suraweera C, de Silva VA (2017). Antipsychotic-associated weight gain: management strategies and impact on treatment adherence. Neuropsychiatr Dis Treat.

[19] Weiden PJ, Mackell JA, McDonnell DD (2004). Obesity as a risk factor for antipsychotic noncompliance. Schizophr Res.

[20] Manschreck TC, Boshes RA (2007). The CATIE schizophrenia trial: results, impact, controversy. Harv Rev Psychiatry.

[21] Lieberman JA, Stroup TS, McEvoy JP, Swartz MS, Rosenheck RA, Perkins DO, Keefe RS, Davis SM, Davis CE, Lebowitz BD, Severe J, Hsiao JK, Clinical Antipsychotic Trials of Intervention Effectiveness (CATIE) Investigators (2005). Effectiveness of antipsychotic drugs in patients with chronic schizophrenia. N Engl J Med.

[22] Kinon BJ, Basson BR, Gilmore JA, Tollefson GD (2001). Long-term olanzapine treatment: weight change and weight-related health factors in schizophrenia. J Clin Psychiatry.

[23] Jónsdóttir H, Opjordsmoen S, Birkenaes AB, Simonsen C, Engh JA, Ringen PA, Vaskinn A, Friis S, Sundet K, Andreassen OA (2013). Predictors of medication adherence in patients with schizophrenia and bipolar disorder. Acta Psychiatr Scand.

[24] Velligan DI, Weiden PJ, Sajatovic M, Scott J, Carpenter D, Ross R, Docherty JP, Expert Consensus Panel on Adherence Problems in Serious and Persistent Mental Illness (2009). The expert consensus guideline series: adherence problems in patients with serious and persistent mental illness. J Clin Psychiatry.

[25] Puhl RM, Himmelstein MS, Pearl RL (2020). Weight stigma as a psychosocial contributor to obesity. Am Psychol.

[26] Seidell JC (1998). Societal and personal costs of obesity. Exp Clin Endocrinol Diabetes.

[27] Lester H, Marshall M, Jones P, Fowler D, Amos T, Khan N, Birchwood M (2011). Views of young people in early intervention services for first-episode psychosis in England. Psychiatr Serv.

[28] Pillinger T, McCutcheon RA, Vano L, Mizuno Y, Arumuham A, Hindley G, Beck K, Natesan S, Efthimiou O, Cipriani A, Howes OD (2020). Comparative effects of 18 antipsychotics on metabolic function in patients with schizophrenia, predictors of metabolic dysregulation, and association with psychopathology: a systematic review and network meta-analysis. Lancet Psychiatry.

[29] Firth J, Siddiqi N, Koyanagi A, Siskind D, Rosenbaum S, Galletly C, Allan S, Caneo C, Carney R, Carvalho AF, Chatterton ML, Correll CU, Curtis J, Gaughran F, Heald A, Hoare E, Jackson SE, Kisely S, Lovell K, Maj M, McGorry PD, Mihalopoulos C, Myles H, O'Donoghue B, Pillinger T, Sarris J, Schuch FB, Shiers D, Smith L, Solmi M, Suetani S, Taylor J, Teasdale SB, Thornicroft G, Torous J, Usherwood T, Vancampfort D, Veronese N, Ward PB, Yung AR, Killackey E, Stubbs B (2019). The Lancet Psychiatry Commission: a blueprint for protecting physical health in people with mental illness. Lancet Psychiatry.

[30] Barton BB, Segger F, Fischer K, Obermeier M, Musil R (2020). Update on weight-gain caused by antipsychotics: a systematic review and meta-analysis. Expert Opin Drug Saf.

[31] Ventriglio A, Gentile A, Stella E, Bellomo A (2013). Metabolic issues in patients affected by schizophrenia: clinical characteristics and medical management. Front Neurosci.

[32] Waite F, Langman A, Mulhall S, Glogowska M, Hartmann-Boyce J, Aveyard P, Lennox B, Kabir T, Freeman D, Oxford Cognitive Approaches to Psychosis Patient Advisory Group (2022). The psychological journey of weight gain in psychosis. Psychol Psychother.

[33] Bak M, Drukker M, Cortenraad S, Vandenberk E, Guloksuz S (2021). Antipsychotics result in more weight gain in antipsychotic naive patients than in patients after antipsychotic switch and weight gain is irrespective of psychiatric diagnosis: a meta-analysis. PLoS ONE.

